# Functional relevance as a principle of translation problem-solving

**DOI:** 10.3389/fpsyg.2022.1073532

**Published:** 2022-12-23

**Authors:** Zhonggang Sang

**Affiliations:** School of Foreign Studies, Xi’an Jiaotong University, Xi’an, Shaanxi, China

**Keywords:** relevance, functionality, interpretive resemblance, translation problem, poem with medicine puns

## Abstract

Translation is both an interpretive use of language and problem-solving activity. In his work, Ernst-August Gutt adopts a Relevance-Theoretic approach to unveil the inferential nature of translation as interpretive language use. He holds that in translating a translator aims to seek the interpretive resemblance between the ST (source text) and the TT (target text). However, Gutt does not explain how interpretive resemblance can be achieved when translation problems arise. Textual function refers to the intended cognitive effects that a text yields on the part of the readers. Considering that it is only when the textual outcome of a translation activity is both relevant and functional that it is a successful interpretive use of language, we propose Functional Relevance as a principle of translation problem-solving. Namely, a translator needs to strategize their solutions to translation problems by making the explicatures and implicatures of the TT resemblant enough both to justify its reader’s processing effort and to fulfill the contextualized textual functions of translation. This can be exemplified by two English translations of Chinese medicine pun poems in a *pien wen*, an archaic literary genre popular in China during the tenth century.

## 1. Gutt’s relevance-theoretic account of translation

Relevance Theory is a cognitive approach to pragmatics and it aims to account for how the meaning is inferred in the actual use of language (Wilson and Sperber, 2002: 45–46). Gutt (2000) uses the theory to investigate translation, which he regards as a type of interlingual interpretive use of language (Gutt, 2000: 105). According to Gutt (2000), the goal of translation is to achieve interpretive resemblance, namely the resemblance between the explicatures and implicatures in the ST and those in the TT (2000: 40). The explicatures refer to propositional enrichments of a logical expression of an utterance (Yus, 1998: 316), they are the information that readers could get by analyzing the text alone; the implicatures are contextual assumptions that readers need to recover in order to meet their expectations (Yus, 1998: 316), they are the information that readers could only get by inferentially analyzing the text together with the context (Gutt, 2000: 40). To realize this goal, a translator either adopts the method of direct translation to make the TT interpretively resemble the ST completely, or selects the method of indirect translation to make the two texts interpretively resemble each other only in relevant aspects (2000: 168–200).

In the past decades, Gutt’s Relevance-Theoretic framework of translation has been widely referenced by translation scholars. However, it does not explain when direct translation or indirect translation is to be employed in translating. Generally, translation is a process of problem-solving (Pym, 2007: 44). Gutt’s theory does not address the topic of translation problem solving. Though he insists that a translator keeps their linguistic choices in line with optimal relevance that is dependent on the target language readers’ processing effort and the cognitive effects produced by the TT (Gutt, 1996: 241–242), he does not answer the question of what choices for a problem-solving translator to make can guarantee that the effects are sufficient and the processing effort is worthwhile.

It has to be pointed out that adopting Gutt’s approach, Yus (2012, 2016), Díaz-Pérez (2013, 2014, 2021a,b: 279–302; 108–129), and Moneva and Angeles (2018) have studied the translation problems related to humor, jokes, puns idiolect, and figures of speech such as irony and parody. Yus (2012, 2016: 117–147; 240–244) holds that the interpretive resemblance between a joke in the ST and a translated one in the TT can be achieved from the cultural, semantic, and pragmatic aspects. The cultural aspect refers to the unity of shared background assumptions between the author and the audience. The semantic one means the linguistic properties of jokes or humor. The pragmatic aspect, outweighing the other two, includes the audience’s inference and the balance between their processing effort and cognitive effects. Díaz-Pérez (2013, 2014: 279; 108) proposes that the selection of strategies in translating puns is governed by the principle of relevance when there is no coincidence between the signifier and the signified across the language boundaries. Namely, a translator will have to evaluate what is more relevant in terms of the content and effect that the puns produce. Moneva and Angeles (2018: 121–144) contends that the translator’s decision-making in dealing with irony and parody is guided by the relevance-theoretical notion of interpretive resemblance, i.e., the TT readers are expected to work out the implicatures similar to those derived from the ST by the ST readers in relevant respects. These studies, though interesting, do not provide a unified account of translation problem-solving mechanism and they touch little upon the social-cultural, situational and conditioning factors constraining translation, a problem-loaded interpretive use of language. Given this, this research aims to account for how a translator makes decisions to cope with translation problems in light of the Relevance Principle when confronted with these constraining factors.

## 2. Context as the strategic resource for translation problem-solving

Context is a hearer-centered concept in Relevance Theory (Monacelli, 2009: 63; Kecskes, 2014: 274–275). According to Sperber and Wilson (1995), context is a cognitive construct and it is a subset of the hearer’s assumptions affecting the interpretation of an utterance (Sperber and Wilson, 1995: 15). It encompasses not only the physical environmental factors or the preceding utterance but also the hearer’s expectations about the utterance (*ibid*: 15–16). A translator, however, is not only a hearer of the ST but also the constructor of the TT and the one who deals with translation problems (Díaz-Pérez, 2014: 108–109). In the process of translation, the major task of a translator is to fill the communicative gap resulting from translation problems (Yus, 2012: 126). Especially, the intentionality of the TT is not always in line with that of the ST as the initiator, commissioner, publisher or client may take part in negotiating the purpose of translation in a specific situation (Nord, 2001: 19).

In translating, what a translator is confronted with are different types of translation problems and heterogeneous factors including the social-cultural, situational and conditioning ones, together with the subjective ones such as the intentions of the initiator and the translator, the expectations of the target readers, and so on. A translation problem can be defined as an objective or inter-subjective task of language transfer facing every translator (regardless of their abilities and technical equipment; Nord, 2006: 166–167). Translation problems can be divided into pragmatic, linguistic, textual, and convention-related ones. Pragmatic problems are caused by the disagreement between the function of the ST (source text) and that of the TT (target text); linguistic problems are brought about by the structural differences between the two languages; textual problems result from the salient features of the ST, which cannot be adequately rendered in the target language; convention-related problems come from the differences of the culture-specific norms and conventions (*ibid*: 174–177). Of course, translation problems may result from the interaction of different factors.

As a rule, a translator is motivated to carry out a translation activity to meet their needs. Translation activity is actualized through goal-directed actions which are finally realized by a series of routinized operational acts (Chesterman, 1997: 89–90). An operational translation act is conditioned by factors including translation problems, the functions, and features of the ST as well as the translator’s habitual translation and language style (Sang, 2018: 132). When the translation act is guided by the translator’s intention, namely their needs objectified in a certain situation, it turns into a translation action constrained by situational factors including interpersonal relationship, place, time, etc. (*ibid*: 131). When the textual outcome of the translation action not only meets the needs of the translator but also those of the commissioner, publisher, and the reader of the TT, it turns into a meaningful social activity. The activity of translation is governed by socio-cultural factors such as translation norms, ideology, ethics, translation laws, and so on (*ibid*: 130). These heterogeneous factors available in the translator’s cognitive environment make up the context of translation. These factors are not static but work dynamically as the cognitive resource for the translator to build the TT (Baker, 2006: 332). The context, in other words, is the strategic resource for translational decision-making (Baker, 2006: 328). As the major part of translational decision-making is to strategize the solutions to translation problems, the context of translation can also be regarded as the strategic resource for translation problem-solving. Admittedly, context is a blurred concept. In the actual use of language, it is crystalized by contextualized textual functions.

## 3. Contextualized textual functions as a basis for translators’ decision-making

Written text-based use of language is an activity in which a motivated language user transforms an idea, thought, attitude, or feelings into a meaningful textual product with the help of other participants such as readers, transmitters, or publishers (Sang, 2019: 543). A language-use activity is meaningful or functional on the condition that the textual outcome meets the expectations and the needs of all the participants. In other words, it is an activity in which a language user externalizes their needs while taking into consideration the socio-cultural, situational, and conditioning factors together with the needs of the other participants. In the process of the activity, the language user follows the prospective textual functions to make linguistic choices (Sang, 2019: 538). Generally, the textual function refers to the cognitive effects or contextual effects that a text or part of a text is intended to yield on the part of the reader in a communicative situation. As for any instance of successful language use, the cognitive effects that its textual outcome yields not only meet the reader’s expectation but also satisfy the needs of the speaker and other participants.

According to Relevance Theory, cognitive effects come from the synthesis of the new and given information, and they are produced when the hearer/reader’s assumptions about an utterance/text are strengthened, confirmed, or eliminated (Sperber and Wilson, 1995: 109; 112). In other words, cognitive effects can be defined as ‘change in one’s awareness’ when there is a crucial interaction between the new and old information (Gutt, 1996: 241–242). As for a well-organized text, the cognitive effects that it yields on the part of the reader can be obtained only by inferring both the text and context (Gutt, 1996: 241). In this sense, the cognitive effects that a text yields precondition its functionality, that is, the greater its cognitive effects, the more relevant it is and the more meaningful or functional it is to the participants.

In the activity of language use, the textual functions that a language user follows are contextualized and hierarchically stratified into the social function and situational function of the TT (at the higher strata) and the conventional function of textual tools (at the basic stratum). When there is any incongruity among these functions, they need to prioritize the one at a higher stratum (Sang, 2019: 538). Social textual function refers to the cognitive effects which not only satisfy the needs of all the participants in the language use activity but also conform to the social rules governing the activity. The situational textual function is the cognitive effects intended by the author to yield on the part of the reader in a certain situation. The conventional function is defined as the cognitive effects that the same genre of text conventionally produces on the part of average readers. The conventional function can also be roughly divided into three types: referential, expressive, and persuasive (Sang, 2019: 545). Generally, a text has quite a few functions but one of them always plays a predominant role (*ibid*: 545). A language user follows the leading textual function to make linguistic choices to build the textual properties, that is, the emphasis of their linguistic choices is laid on the content of a text whose major function is to produce mainly referential or informative effects, on the form of a text whose dominant function is expressive, and on the appealing effects of a text whose function is mainly persuasive (Reiss, 2000: 26). For example, the function of a literary text is expressive and the author usually focuses the linguistic choices on the formal textual features producing artistic and aesthetic effects (Reiss, 2000: 34). As for politicians’ public speeches, their main function is to appeal to the audience for their support, the language users would prioritize their linguistic choices that may yield a persuasive effect. Added to that, as each textual component may play a different structural role to fulfill the global function of the text, the structural importance of the textual component needs to be assessed in making choices.

Translation, as Gutt (1996) points out, is a type of interpretive language use (Gutt, 1996: 251). During the process of this language use, a translator makes decisions by the contextualized textual functions of the prospective TT which crystallizes not only the subjective factors such as the author’s intention, the readers’ expectation, but also the socio-cultural, situational, and conditioning factors ones (Nord, 2006: 77). The contextualized textual functions in a translation activity are hierarchically stratified into the social function and situational function of translation as well as the function of the ST. If there is any disagreement among them, a translator would prioritize the one at a higher stratum (Sang, 2018: 134–135). The social function of translation refers to the cognitive effects that not only meet the needs of the translator, commissioner, publisher, and the TT readers but also comply with the social rules governing the translation activity. The situational function of translation is the cognitive effects that the TT is intended by the translator to produce on the target language readers in a specific situation. When there is any disagreement among the contextualized textual functions, a translator would prioritize the social function of translation over its situational function, which they would put ahead of the function of the ST (Sang, 2018: 130–131). For example, if a translator is commissioned to translate a classified document on a new scientific invention without the authorization of the inventor, the referential cognitive effects that the TT is intended to produce are illegitimate as the translation action breaches the Law of Intellectual Property Rights, one of the social rules governing the translation activity. In this case, the translator has to base their decisions on the social function of translation and re-negotiate with the commissioner or the initiator.

Translation is essentially a process of problem-solving. Contextualized textual functions, of course, are also one of the strategic bases of translation problem-solving. For instance, to acquaint himself with the Chinese history of the Northern Dynasties, a French historian commissions a translator to translate from Chinese into French the classic narrative poem *The Song of Mu Lan* (木蘭辭) which was based on a legend of the historic period. There arises a pragmatic translation problem as the ST is a literary text whose function is mainly expressive (i.e., to produce the artistic and aesthetic cognitive effects), but the cognitive effects that the TT is expected to yield are largely referential and informative. To solve this problem, the translator would prioritize the textual function of translation over that of the ST and they may choose the linguistic means that ensure the intelligibility of the TT content at the sacrifice of the formal features of the ST.

Admittedly, textual functions are by definition the intended cognitive effects that the textual outcome of a language use activity produces on the part of the reader. Although contextualized textual functions serve as a basis of a language user’s decision-making or a translator’s problem-solving, they are not helpful to spell out how the cognitive effects are produced on the part of the reader. This is where the Principle of Relevance comes into play.

## 4. Functional relevance as a principle of translation problem-solving

As discussed above, the context of translation is the strategic resource to handle translation problems. To be specific, a translator strategizes their solutions to translation problems by the hierarchical contextualized textual functions which not only embody the subjective factors such as the needs and the expectations of the participants, but also the socio-cultural, situational, and conditioning factors. As textual function is identified as the cognitive effects that a text is intended to produce on the part of the reader, the greater the cognitive effects that the TT yields, the more relevant it is to the target readers and the more functional or meaningful the translation activity is to all the participants.

The Principle of Relevance highlights the equilibrium between cognitive effects and processing effort. As Sperber and Wilson (1995) points out, relevance is a matter of degree (1995: 123). Maximal relevance means that the greatest cognitive effects are obtained at the cost of the lowest processing effort. Optimal relevance refers to the fact that the hearer’s expended processing effort is justified by adequate cognitive effects (*ibid*: 270). The quantity of cognitive effects and processing effort, however, does not qualify the cognitive effects as what the participants of a language use activity need or expect. For instance, William Carlos Williams’s poem ‘This is Just to Say’ (*This just to say/I have eaten/the plums/that were in/the icebox/and which/you were probably/saving for breakfast/Forgive me/they were delicious/so sweet/and so cold*) can either be translated into a daily note or a literary text (Arrojo, 2005: 239–242). If it is translated to be included in an anthology of literature, the version of daily note will not be functional or meaningful at all even though it can yield greater cognitive effects at the cost of lower processing effort.

Relevance theorists make a distinction between descriptive language use and interpretive language use. An utterance is a descriptive use of language when its propositional form is true of the state of affairs. An utterance is an interpretive use of language when it is intended to represent what someone else said or thought (Sperber and Wilson, 1995: 228–231). Translation, from a Relevance Theory Perspective, is an interlingual interpretive use of language (Gutt, 2004: 105). The TT is the product of interpretive language use as it interpretively resembles the ST. Interpretive resemblance, which depends on the number of explicatures and implicatures that the two texts share, is the criterion of translation (Díaz-Pérez, 2014: 123). In translating, a translator aims at either achieving the complete interpretive resemblance between the ST and the TT or making the explicatures and implicatures in the TT adequately resemble those in the ST in relevant respects (Gutt, 2004: 169–171).

Interpretive resemblance, however, does not guarantee that a translation is functional. Taking the Chinese-French translation of *The Song of Mu Lan* (木蘭辭) for another example, the formal features of the ST weigh heavily in producing implicatures. However, if the textual functions of translation have not been taken into consideration, no matter how much resemblance of the implicatures is achieved, and no matter how great the cognitive effects the TT yields, it is likely that the translation still will not be meaningful. This is because what the commissioner and the French historian expect is mainly intelligible historic information, not the resemblant implicatures in the TT. In fact, the formal features related to the rhyme scheme of the ST are impossible to be adequately rendered in the target language. If a translator goes all out to achieve the resemblance of the implicatures derived from these features, she has but to sacrifice the intelligibility of the TT.

**Figure 1:**
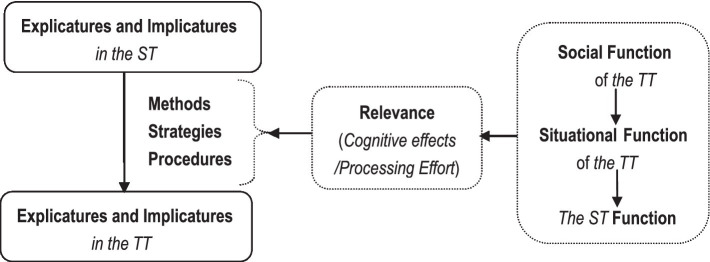
Mechanism of decision-making in translating.

Translation is a meaningful interpretive use of language if and only if the processing effort that the TT costs is justified by the adequate cognitive effects which fulfill the hierarchically contextualized textual functions (i.e., the social translation function, the situational translation function, and the function of the ST). In other words, it is only when the textual outcome is both relevant and functional that the translation activity is a successful inter-lingual interpretive use of language. In translating, therefore, a translator needs to aim to build a TT with adequate explictures and implicatures that are of optimal relevance and functionality. Their choices of translation methods, strategies to solve translation problems, and linguistic procedures to construct the TT need to conform to the Principle of Relevance and contextualized textual functions. This can be illustrated as follows,

As shown in Figure 1, a translator chooses the methods, strategies, and procedures to ensure that the TT and the ST share as many explicatures and implicatures as possible. Translation method embodies the superordinate goal of translation activity and it is the global plan for the whole process of the interpretive use of language (Molina et al., 2002: 508). Translation strategy refers to the conscious plan to tackle translation problems (Zabalbeascoa, 2000: 120). Translation procedures are the linguistic means or techniques employed to build the TT. Translation method and strategy are realized through translation procedures.

Given the translation problems resulting from the linguistic, cultural, textual, pragmatic, and convention-related differences between the ST and the TT, it is not always possible to make the explicatures and implicatures of the TT completely resemble those of the ST. At this point, a translator would have to select the strategies to guarantee that the cognitive effects not only justify all the TT reader’s processing effort but also conform to the hierarchically contextualized textual functions. If there is any disagreement among them, the textual function at a higher stratum, namely social function or situational function of translation needs to be prioritized.

**Figure 2:**
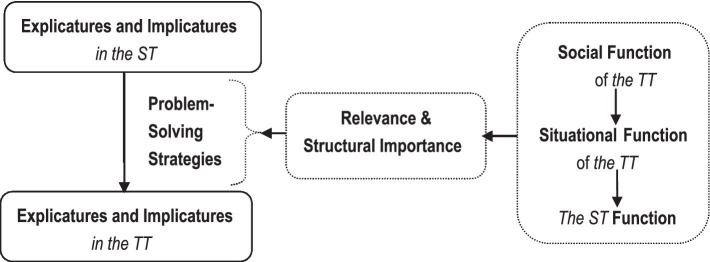
Functional relevance as principle of translation problem-solving.

As translation problems rest on the textual components of specific structural importance to the global textual function, the problems of the same type in the same text may be tackled differently due to their structural roles in shaping the global textual function. Based on this, how translation problems are solved can be further explained as follows,

As illustrated in Figure 2, in translating it is not always the case that the explicatures and implicatures of the TT can be made to resemble those of the ST completely as translation problems get in the way. To tackle the problems attached to specific textual components, a translator needs to select the strategies and procedures to guarantee that the explicatures and implicatures of the TT not only are resemblant enough to justify all the TT reader’s processing effort but also conform with the structural importance to the contextualized textual functions. This is the principle of Functional Relevance, which governs the way a translator selects strategies to solve translation problems.

## 5. Exemplification: Two English translations of a Chinese poem with medicine puns in a *pien wen*

Functional Relevance as a principle of translation problem-solving can be exemplified by two English translations of a Chinese poem with medicine puns in a *pien wen*. Tun-Huang manuscripts are ancient Chinese documents that were unearthed in the Mogao grottos of Tun-Huang in northwest China in the early 1900s. Among the manuscripts are bundles of literary texts labeled as *pien wen* (變文). *Pien wen* is a literary genre of popular narrative widely employed in the tenth century of China. *Pien wen* is written in prosimetric style (i.e., a combination of prose and verse) and the language is semicolloquial (Mair, 1983: 5). There is no doubt that *pien wen* itself may pose a convention-related translation problem in translating.

Arthur Waley translated *Wu Tzu-hsü* (伍子胥變文), a typical piece of *pien wen*, into English in 1960. Victor H. Mair re-translated it in 1983. This piece of *pien wen* is centered on Wu Tzu-hsü, a historic figure who lived in the late sixth and early fifth century B.C. The story begins with an incident: the King of Ch’u was annoyed by Wu She, Wu Tzu-hsü’s father, for he criticized the king for having married his son’s fiancée. The king incriminated the whole family of Wu She and Wu Tzu-hsü became a fugitive. During his exile, Wu Tzu-hsü managed to meet his long-waiting wife. To conceal the identity of being a fugitive from the passerby, the couple pretended to be strangers by conversing in the form of a poem with Chinese medicine puns, which is one of the traditional genres of classical Chinese poetry. The poem of this genre sounds like a verse made up of the names of herbs and minerals used in Chinese medicine. It can be interpreted either in terms of the explicatures, namely the referential meaning of the medicine names, or the implicatures derived from these names or their **homophones.** In most cases, what is intended to express is none other than the implicatures, built through an inferential synthesis of the text and the context. In the *pien wen*, the wife of Wu Tzu-hsü produced a poem whose first stanza goes as follows,

其妻遂作藥名詩問曰:妾是***仵茄***之婦***細辛***，早***仕於梁***，就禮未及***當歸***，使妾閒居***獨活***。***蒿莨薑芥***，*澤瀉*無鄰；仰歎***檳榔***，何時***遠志***。(Wang, 1957: 10; Italicized for emphasis).

The excerpt above involves 11 Chinese medicine names: ‘仵茄’, ‘細辛’, ‘仕於梁’ (‘禹餘糧’), ‘當歸’, ‘獨活’, ‘蒿’, ‘莨薑’, ‘芥’, ‘澤瀉’, ‘檳榔’, ‘遠志’. Their scientific names are ‘Eleutherococcus brachypus’, ‘Asarum sieboldii’, ‘Limonitum’, ‘Angelica’, ‘Radix Angelicae pubescentis’, ‘Artemisia’, ‘Chinese galangal’, ‘Brassica juncea’, ‘Rhizoma alismatis’, ‘Areca catechu’, and ‘Polygala tenuifolia’. Among these medicine names, the **homophone** of ‘仵茄’ is ‘伍家’ (the Wu family). The homophone of ‘細辛’ is ‘媳新’ (a newlywed wife). “仵茄之婦細辛” suggests that she was a newlywed wife of the Wu family. ‘仕於梁’ (word for word translation: working as an official in Liang) suggests that Wu Tzu-hsü served in the court of the Kingdom of Liang. ‘就禮’ means ‘consummating marriage’ in Chinese and ‘當歸’ means ‘that he should go back’. ‘就禮未及當歸’ implies that Wu Tzu-hsü had to go back before his wedding could be consummated. The word for word translation of ‘獨活’ is ‘living alone’. ‘使妾閒居獨活’ means that Wu Tzu-hsü left his wife alone at home.

The sentence ‘蒿 (wormwood) 莨薑 (galangal) 芥 (mustard)’ means that the yard and the field were blanketed by overgrown weeds. ‘宅歇’ (word for word translation: living in a house) is the homophone of ‘澤瀉’ (Alisma). ‘無鄰’ means ‘without neighbors’ in Chinese. By ‘澤瀉無鄰’, there are hints that the wife lived in a house without neighbors. The homophone of ‘檳榔’ (betel nut) is ‘賓郎’ which means ‘her husband working in a country other than his motherland’. ‘仰歎檳榔’ can be interpreted as ‘I raise my head and sigh for the sake of my husband who works in a country other than his motherland’. ‘何時遠志’ means ‘remembering me whenever you are faraway’ in Chinese. These implicatures could only be inferred by combining the text with the context.

A pun is a figure of speech that a special rhetorical effect is produced by using one word whose homophone or homonym may elicit two different well-matched meanings. As there is always no equivalence between the homophone or homonym of a word in one language and that in another, the puns in the ST, to some extent, are untranslatable and they bring about a translation problem. The poem of Chinese medicine puns, undoubtedly, poses a textual translation problem as it is a salient textual feature impossible to be adequately rendered in the target language. The English words for these Chinese medicine names or their homophonies are not helpful at all to build the above-mentioned implicatures. To deal with **this** translation problem, Waley (1960) inserted a narratorial commentary explicitly explaining the poem of Chinese medicine puns as follows,

The wife then shows that she knows who he is and what has been happening to him in a passage consisting largely of the names of medicines, both vegetable and mineral, used punningly. For example, tang-kuei means a kind of angelica, but also ‘you must go back’. Tu-huo means another kind of angelica, but also ‘live alone’. Wu Tzu-hsü replies in the same vein. This passage is of course untranslatable, as the plays on words cannot be reproduced in English. (Waley, 1960: 35-36)

The commentary gives an account of what the poem is about, what features it has, and why it was not fully translated, that is, the plays on words in the ST are ‘untranslatable’. The implicatures related to the Chinese medicine puns were made into the explicatures in the TT.

As previously discussed, Wu Tzu-hsü is a literary text whose function is mainly expressive, that is, to produce artistic and aesthetic cognitive effects on the part of the reader (*cf.* Reiss, 2000: 34). As a rule, the formal features of a literary text are purely intentional and they weigh heavily in a translator’s decision-making (Sang, 2006: 48; 50). However, very little was known to English readers about the manuscript of *pien we*n in the 1960s and it was the first time that *pien wen* was translated into English (Waley, 1960: 238). The English readers, at that time, were expecting the information about the story itself more than the artistic and aesthetic values of the newly discovered manuscript. Given the vast amount of translation problems caused by the linguistic, textual, and cultural differences, Waley (1960) estimated that the contextualized textual function of translation was largely informative rather than expressive. He, therefore, changed the expressive textual function into the informative one and intended his translation for the general readers (1960: 238). To ensure that they could get adequate information about the story content at the cost of unnecessary processing effort, he avoided ‘discussion of linguistic and textual problems’ to a large extent (Waley, 1960: 238–239). Though the poem is of high structural importance to the expressive function of a literary text, its role will be decreased in the TT whose social function is made largely informative. This is why he put in the explanatory commentary above to handle the translation problem. In other words, to solve the textual translation problem caused by the poem of Chinese medicine puns, the translator made the implicatures into explicatures to keep the cognitive effects in line with the structural importance to the contextualized textual functions and the general readers could get enough positive effects without taking too much processing effort.

Twenty-three years later, Mair (1983) retranslated the poem in *Wu Tzu-hsü* as follows,

I, Belladonna, am the wife of a man named Wahoo, / who early became a mandrake in Liang. / Before our matrimony vine could be consomméted, he had to go back, / Leaving me, his wife, to dwell here ruefully alone. / The mustard has not been cut, the flaxseed bed remains unvisited- / Hemlocked in here without any neighbors, I raised my head and / sighed for my Traveler's Joy: / “Parsley, sage, rosemary, and thyme- / I pray that he'll forget me not!” / (Mair, 1983: 135)

It is noteworthy that up to 1960 when Arthur Waley translated the *pien wen* from Chinese into English, little research work had been done on the Tun-Huang manuscripts though Marc Auriel Stein and Paul Pelliot took thousands of them to London and Paris in the early 20th century (Waley, 1960: 238). However, in the year 1983 when Victor H. Mair re-translated the *pien wen*, there was a great development in the international studies on the Tun-Huang manuscripts which attracted an increasing number of English readers specializing in sinology and Chinese literature. There is no doubt that the contextualized functions of Mair’s re-translation are different from those of Waley’s ‘debut translation’. The participants of the translation activity including the author, the publisher, and the specialist readers expected that the TT could produce the same artistic and aesthetic effects as the source literary text, and the explicatures and implicatures of the TT could resemble those of the ST as much as possible.

As shown above, the first stanza of Mair’s translation also includes 13 names of medicinal herbs: ‘belladonna’, ‘wahoo’, ‘mandrake’, ‘matrimony vine’, ‘mustard’, ‘flaxseed’, ‘hemlock’, ‘traveler’s joy’, ‘forget me not’, ‘parsley’, ‘sage’, ‘rosemary’, and ‘thyme’. Although there is no one-to-one correspondence between the Chinese and English herbal names which can be used in the same punning way, these names in the TT enable it to resemble the ST in terms of the explicatures and implicatures as well as the textual features of the poem with medicine puns. ‘Belladonna’ is not only the name of a medicinal herb but also the given name of a woman. ‘Wahoo’, the homophone of ‘伍侯’ (i.e., Lord of Wu), refers to Wu Tzu-hsü. ‘Mandrake’ sounds like ‘mandarin’ (senior official), which implies that Wu Tzu-hsü was a senior official in the kingdom of Liang. In ‘matrimony vine’, ‘matrimony’ means marriage. The sentence that ‘before our matrimony vine could be consomméted, he had to go back’ implies that before they could consummate their marriage, Wu Tzu-hsü had to go back to Liang. ‘Hemlocked’ sounds like ‘home-locked’, suggesting that the wife locked herself at home and lived alone without neighbors. In ‘sighed for my Traveler’s Joy’, ‘Traveler’s Joy’ could not only be understood as a garden plant but also as the ‘traveler’ Wu Tzu-hsü who brought his wife joy. ‘Forget me not’ can be considered both as a flowering plant (i.e., Myosotis sylvatica) and as the wife’s praying that her traveler husband would ‘forget her not’. It is noteworthy that ‘parsley, sage, rosemary, and thyme’ happen to be the words of ‘Scarborough Fair’, an England folk song which may remind the TT readers of the implicatures about Wu Tzu-hsü wife’s praying as the following line in the song is ‘Remember me to one who lives there’. There is no doubt that the implicatures could only be built by interpreting the herbal names (their homophonies or the words that sound alike) together with the context.

As discussed above, *Wu Tzu-hsü* is a literary text whose major function is expressive. This is identical to the contextualized function of Mair’s translation, which was expected to produce resemblant aesthetic and artistic cognitive effects. It is clear that the poem of medicine puns, which poses a textual translation problem, is of great structural importance to fulfill the function of translation. To solve this problem, the translator went all out to restructure an English poem with the punning names of medicinal herbs. The explicatures and implicatures resembling those of the ST could be derived in the same vein. Though the readers of Victor Mair’s translation, compared with those of Arthur Waley’s, needed to expend more effort to process the poem of medicine puns, they could get the adequate cognitive effects they wished for.

Generally, translation problems rest on the specific textual components of the ST. As exemplified by the translated Chinese poem of medicine puns, the translators strategized their solutions to the textual problem by making the TT adequately relevant to the target readers and interpretively resembling the ST in light of the structural importance of the textual component to the translation function. In other words, the extent to which the ST interpretively resembles the TT in relevant respects is dependent on the structural importance to contextualized functions of translation.

## 6. Conclusion

Translation is an interpretive use of language. Ideally, a translated text and its source text are thought to share all the explicatures and implicatures. However, given the linguistic, textual, pragmatic, or convention-related translation problems, the interpretive resemblance can only be achieved both in relevant and functional respects, that is to say, a translator needs to strategize their solutions by making the explicatures and implicatures of the TT resemblant enough both to justify the TT reader’s processing effort and to fulfill the contextualized textual functions of translation. Functional Relevance, in this sense, can be termed as a principle for a translator to select strategies to solve translation problems. As for the choice of a translation method, it is dependent on contextualized textual functions. If the function of the TT agrees with that of the ST, the translator would choose the method of direct translation. Otherwise, they would select the method of indirect translation.

Additionally, Functional Relevance may also be true of other written text-based language uses. Namely, the users’ linguistic choices to build the explicatures or implicatures into the TT are not only dependent on their estimated balance between the reader’s processing effort and the positive cognitive effects, but also on how importantly their choices contribute to the contextualized hierarchical textual functions (i.e., the social function and situational function of the TT and the conventional function of textual tools).

## Data availability statement

The original contributions presented in the study are included in the article/supplementary material, further inquiries can be directed to the corresponding author.

## Author contributions

The author confirms being the sole contributor of this work and has approved it for publication.

## Funding

This research was supported by National Social Science Foundation of China (Grant No. 20AZD129).

## Conflict of interest

The author declares that the research was conducted in the absence of any commercial or financial relationships that could be construed as a potential conflict of interest.

## Publisher’s note

All claims expressed in this article are solely those of the authors and do not necessarily represent those of their affiliated organizations, or those of the publisher, the editors and the reviewers. Any product that may be evaluated in this article, or claim that may be made by its manufacturer, is not guaranteed or endorsed by the publisher.
